# Supplementary material for: The Transcriptional Adaptor Protein ADA3a Modulates Flowering of Arabidopsis thaliana
Source: Cells. 2021 Apr 14;10(4):904. doi: 10.3390/cells10040904 (PMC8071052; doi:10.3390/cells10040904)
Supplement: Supplementary file 1 [file cells-10-00904-s001.zip › 2021-04-11-ada3-manuscript-supplementary-revised.docx]

**The transcriptional adaptor protein ADA3a modulates flowering of *Arabidopsis thaliana***

Stylianos Poulios^1^, Despoina Dadarou^1,3^, Maxim Gavriilidis^1,2^, Niki Mougiou^1^, Nestor Kargios^1^, Vasileia Malliori^1^, Amy Hark^4^, John H Doonan^3^ and Konstantinos E.Vlachonasios^1*^

**Supplementary material**

Table S1. Oligonucleotide sequence of primers used

| **Gene** | **Primer name** | **Nucleotide sequence (5’-3’)** | **Experiment** |
| --- | --- | --- | --- |
|  | LBa1 | TGGTTCACGTAGTGGGCCATCG | Genotyping |
| ADA3a | KVJD34-ADA3A_F1 | TGCAGATTATGGTTTCAAGGGTTCA | RT-PCR & Genotyping |
|  | KVJD35-ADA3A_R1 | TCAATCGGGGACGGAGTGTT |  |
|  | KVJD36-ADA3A_B_F2 | GGAATGTCAGCACCTGGGAAGT |  |
|  | KVJD37-ADA3A_B_R2 | CCTGGCCTTCACATTGTTTTC |  |
|  | KVJD38-ADA3A_F3 | TCAAATGATGGTCGTGAGGA |  |
|  | KVJD39-ADA3A_R3 | TTCCAGCCTTTCATTGTTCC |  |
|  | KVJD40-ADA3A_F4 | TGGAGGAGGCTATCTGCAAC |  |
|  | KVJD41-ADA3A_R4 | CGATGGCAGGCTTTAGTAGC |  |
| ADA3b | KVJD42-ADA3B-F1 | TGGAGCGCTACTTTTGCTTT |  |
|  | KVJD43-ADA3B-R2 | ACTCAACATCGGCTCCAAAT |  |
|  | KVJD44-ADA3B-F3 | ATATCATCATTGCCGGGTTG |  |
|  | KVJD45-ADA3B-R3 | TAGTGGTTTCCCACCCACAT |  |
| PDF | KB471-PDF2F | TAACGTCGCCAAAATGATGC | RT-PCR |
|  | KB472-PDF2R | GTTCTCCACAACCGCCTTGGT |  |
| *At4G26410* |  | GAGCTGAAGTGGCTTCCATGA |  |
|  |  | GGTCCGACATACCCATGATCC |  |
| FT | KVDW035-FT-F | CCCTGCTACAACTGGAACAAC | Gene Expression |
|  | KVDW036-FT-R | CACCCTGGTGCATACACTG |  |
| SOC1 | KVDW047-SOC1-F | ACGAGAAGCTCTCTGAAAAG |  |
|  | KVDW048-SOC1-R | GAACAAGGTAACCCAATGAAC |  |
| FLC | KVDW032-FLC-F | TGTGAGTATCGATGCTCTTGTTCA |  |
|  | KVDW033-FLC-R | TTCAACATGAGTTCGGTCTTCTTG |  |
| SPL3 | KVDW041-SPL3-F | CTCATGTTCGGATCTCTGGTC |  |
|  | KVDW042-SPL3-R | TTTCCGCCTTCTCTCGTTGTG |  |
| SPL5 | KVDW045-SPL5-F | AAGGCATCTGCTGCGACTGTTG |  |
|  | KVDW046-SPL5-R | TCCTCCTCCTCTCATTGTGTCC |  |
| SPL9 | KVDW077-SPL9-F | CAAGGTTCAGTTGGTGGAGGA |  |
|  | KVDW078-SPL9-R | TGAAGAAGCTCGCCATGTATTG |  |
| RGA | KVDW049-RGA-F | TACATCGACTTCGACGGGTA |  |
|  | KVDW050-RGA-R | GTTGTCGTCACCGTCGTTC |  |
| GAI | KVDW055-GAI-F | AACTCGGCATGTTGTCCTG |  |
|  | KVDW056-GAI-R | AAAGCGCGTGAACGAGAC |  |
| FCA | KVI126-FCAF | CCCAGACCCAATTACAGCCG |  |
|  | KVI127-FCAR | CTGGCCCCTGGAGTTGGATA |  |
| TOE1 | KVDW059-TOE1-F | CGAGTTATAATAATCCCGCCCGAG |  |
|  | KVDW060-TOE1-R | TTAAGGGTGTGGATAAAAGT |  |
| TOE2 | KVDW061-TOE2-F | ATGGAGAACCACATGGCTGC |  |
|  | KVDW062-TOE2-R | GGTGCTGTAGCTGCTACGGC |  |
| SMZ | KVDW065-SMZ-F | AGGGAGAAGGAGCCATGAAGTTTGGTG |  |
|  | KVDW066-SMZ-R | GTCTTCAGAGGTTTCATGGTTGCCATG |  |
| SNZ | KVDW067-SNZ-F | CAGCAGATTATTACATGGGTTTG |  |
|  | KVDW068-SNZ-R | GGTTTAATTTCTGTGATCGGTAGA |  |
| CDF1 | KVDW087-CDF1-F | AAGCTCTTTGGTATGAAAATTCCTTT |  |
|  | KVDW088-CDF1-R | GTTCCTGTCTTGTTTTGGTTCTTTTCTTC |  |
| SMZ | KVANA53-SMZProF | AACACGTCCCACGTTCTTTT | ChIP |
|  | KVANA54-SMZProR | GCGAGAGAGAGAGGGAGAGA |  |
|  | KVANA55-SMZORFF | TAGGCGAGAGAGTGCGAGTT |  |
|  | KVANA56-SMZORFR | TGAATTGGGTGCATTTTTGA |  |
| SPL3 | KVANA61-SPL3ProF | TCTCGCGACAAAATGTTCAC |  |
|  | KVANA62-SPL3ProR | CTATTTCACCATGCGGGTTC |  |
|  | KVANA63-SPL3ORFF | GGCTTTGGAGAAGAAGCAGA |  |
|  | KVANA64-SPL3ORFR | GGCATGAAACTGGCAGACTT |  |
|  | KVANA65-SPL3DoF | CAGGGATCTGTATTTGTTTTGC |  |
|  | KVANA66-SPL3DoR | ATCACGAATCAAAAGTGTCTCC |  |
| SPL5 | KVANA69-SPL5ProF | AGGAAACGCCCATACTTGTG |  |
|  | KVANA70-SPL5ProR | GCAGACTGCAGATCCCTTTC |  |
|  | KVANA71-SPL5ORFF | TTGTCAACAATGCAGCAGGT |  |
|  | KVANA72-SPL5ORFR | TGAAACCTGCTCAAAACCATC |  |
|  | KVANA73-SPL5DoF | ACGGCTTCTTGGTTTGACAT |  |
|  | KVANA74-SPL5DoR | TGATGGACTATGGAAGGTTTTG |  |
| CDF1 | KVANA77-CDF1ProF | TCCTCGTAGGAGCACTTCGT |  |
|  | KVANA78-CDF1ProR | TCCGTTAACCAAACAGATCAAA |  |
| RGA | KVANA85-RGAProF | CCCAAACCCATTTGCTCTTA |  |
|  | KVANA86-RGAProR | TTTGGGGATGATGAAGGAAG |  |
| GAI | KVANA93-GAIProF | GGTGTGTGTGTGATTTTCAGC |  |
|  | KVANA94-GAIProR | CCGATGAATCATGGATCAGA |  |
| pGADT7-ADA3a | KVI134-5-pGA-NdeI-Ada3a | acgtaccagattacgctcatatgatgtcagcacctgg | Cloning for Y2H assays |
|  | KVI135-3-Ada3a-BamHI-Pga | gcagctcgagctcgatggatcctcataactttatgt |  |
| pGADT7-ADA3b | 5'-pGADT7-NdeI-Ada3b | cgtaccagattacgctcatatgatgtcagcacctg |  |
|  | 3'-ada3b-BamHI-pGADT7 | cgagctcgatggatccttataccatcatgtttaagtctg |  |
| pGADT7-SGF11 | 5'-pGADT7-NdeI-sgf11 | cgtaccagattacgctcatatgatgtctggcgcagaggata |  |
|  | 3'-sgf11-BamHI-pGADT7 | ctcgagctcgatggatcctcagtctcctttcacgttctc |  |
| pGADT7-SGF29a | 5'-pGADT7-NdeI-sgf29a | taccagattacgctcatatgatgtcgtcgtcgccggac |  |
|  | 3'-sgf29a-BamHI-pGADT7 | cgagctcgatggatccttactggcgatggccttctg |  |
| pGADT7-SGF29b | 5'-pGADT7-NdeI-sgf29b | cgtaccagattacgctcatatgatgtcgtcaccagacattgt |  |
|  | 3'-sgf29b-BamHI-pGADT7 | gagctcgatggatcctcactggcgatgaccttctg |  |
| pGADT7-TAF12b | 5'-pGADT7-NdeI-taf12b | taccagattacgctcatatgatggcggaaccgatt |  |
|  | 3'-taf12b-BamHI-pGADT7 | cgagctcgatggatccttagtatcgtgtcatgtgttgtaa |  |
| pGBKT7-ADA3a | KVI136-5-pGB-NdeI-ada3a | tcagaggaggacctgcatatgatgtcagcacctgg |  |
|  | KVI137-3-Ada3a-BamHI-Pgb | gccgctgcaggtcgacggatcctcataactttatgt |  |
| pGBKT7-ADA3b | 5'-pGBKT7-NdeI-ada3b | cagaggaggacctgcatatgatgtcagcacctggga |  |
|  | 3'-ada3b-BamHI-pGBKT7 | cgctgcaggtcgacggatccttataccatcatgtttaagtctg |  |
| pGBKT7-SGF11 | 5'-pGBKT7-NdeI-sgf11 | cagaggaggacctgcatatgatgtctggcgcagaggataa |  |
|  | 3'-sgf11-BamHI-pGBKT7 | gcaggtcgacggatcctcagtctcctttcacgttctctc |  |
| pGBKT7-SGF29a | 5'-pGBKT7-NdeI-sgf29a | cagaggaggacctgcatatgatgtcgtcgtcgccggac |  |
|  | 3'-sgf29a-BamHI-pGBKT7 | gcaggtcgacggatccttactggcgatggccttctgg |  |
| pGBKT7-SGF29b | 5'-pGBKT7-NdeI-sgf29b | tcagaggaggacctg catatg atgtcgtcaccagacattgt |  |
|  | 3'-sgf29b-BamHI-pGBKT7 | ctgcaggtcgacggatcctcactggcgatgacctt |  |
| pGBKT7-TAF12b | 5'-pGBKT7-NdeI-taf12b | agaggaggacctgcatatgatggcggaaccgattccctc |  |
|  | 3'-taf12b-BamHI-pGBKT7 | gctgcaggtcgacggatccttagtatcgtgtcatgtgttgta |  |
| pGBKT7-ADA2a | 5'-pGBKT7-NdeI-ada2a | tgatctcagaggaggacctgcatatgggtcgttcgaa |  |
|  | 3'-ada2a-BamHI-pGBKT7 | tgcaggtcgacggatcctcatgttgagtcacctattcc |  |
| pGBKT7-ADA2b | 5'-pGBKT7-NdeI-ada2b | tgatctcagaggaggacctgcatatgggtcgctctcgag |  |
|  | 3'-ada2b-BamHI-pGBKT7 | gctgcaggtcgacggatccttaaagttgagcaataccc |  |

STable 2. Flowering in *ada3a-1* and *ada3b-1* mutants.

| **Genotype** | **Days to bolting (±SD)** | **P** | **Rosette Leaves (±SD)** | **P** | **N** |
| --- | --- | --- | --- | --- | --- |
| Col-0 | 18.75±1.37 |  | 11.35±1.42 |  | 20 |
| *ada3a-1* | 16.78±1.27 | <0.0001 | 9.15±1.46 | <0.0001 | 19 |
| *ada3b-1* | 17.65±1.78 | 0.0347 | 10.65±1.49 | 0.1366 | 20 |


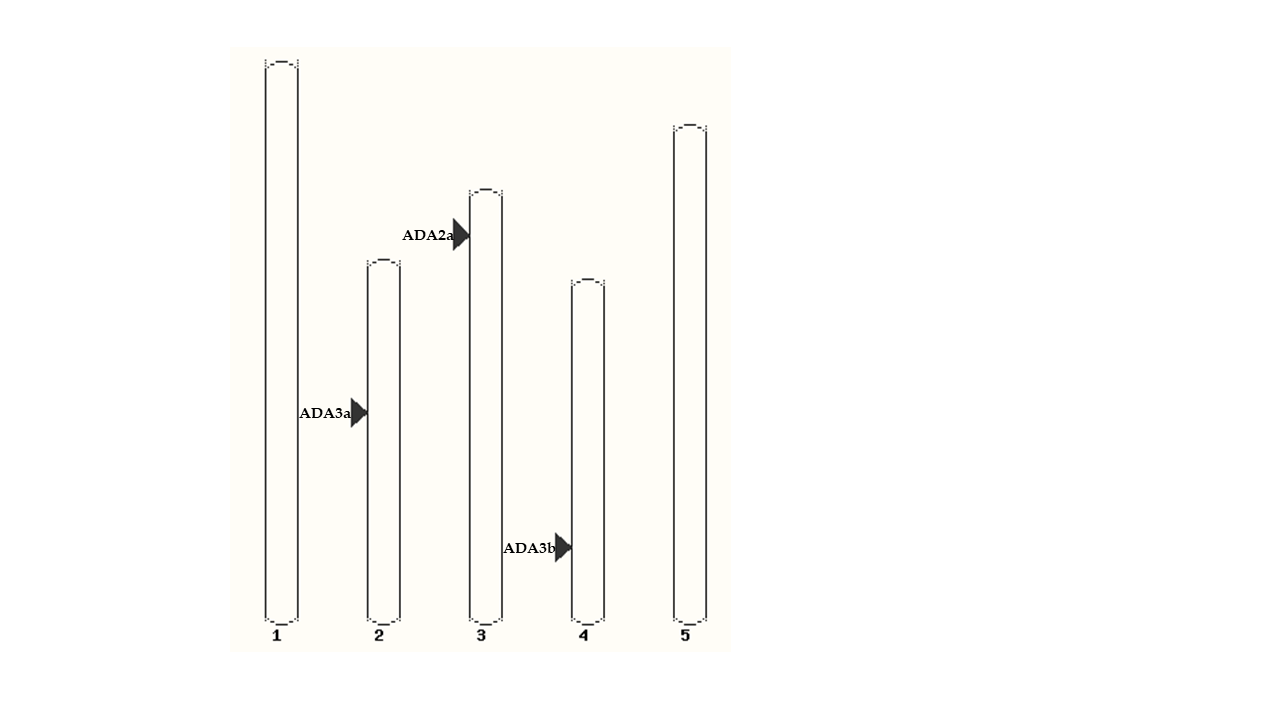
Figure S1. Physical map of ADA2a, ADA3a and ADA3b. Location of genes, ADA2a (At3g07740), ADA3a (At2g19390) and ADA3b (At4g29790) in Arabidopsis genome. Numbers indicate the chromosome number.


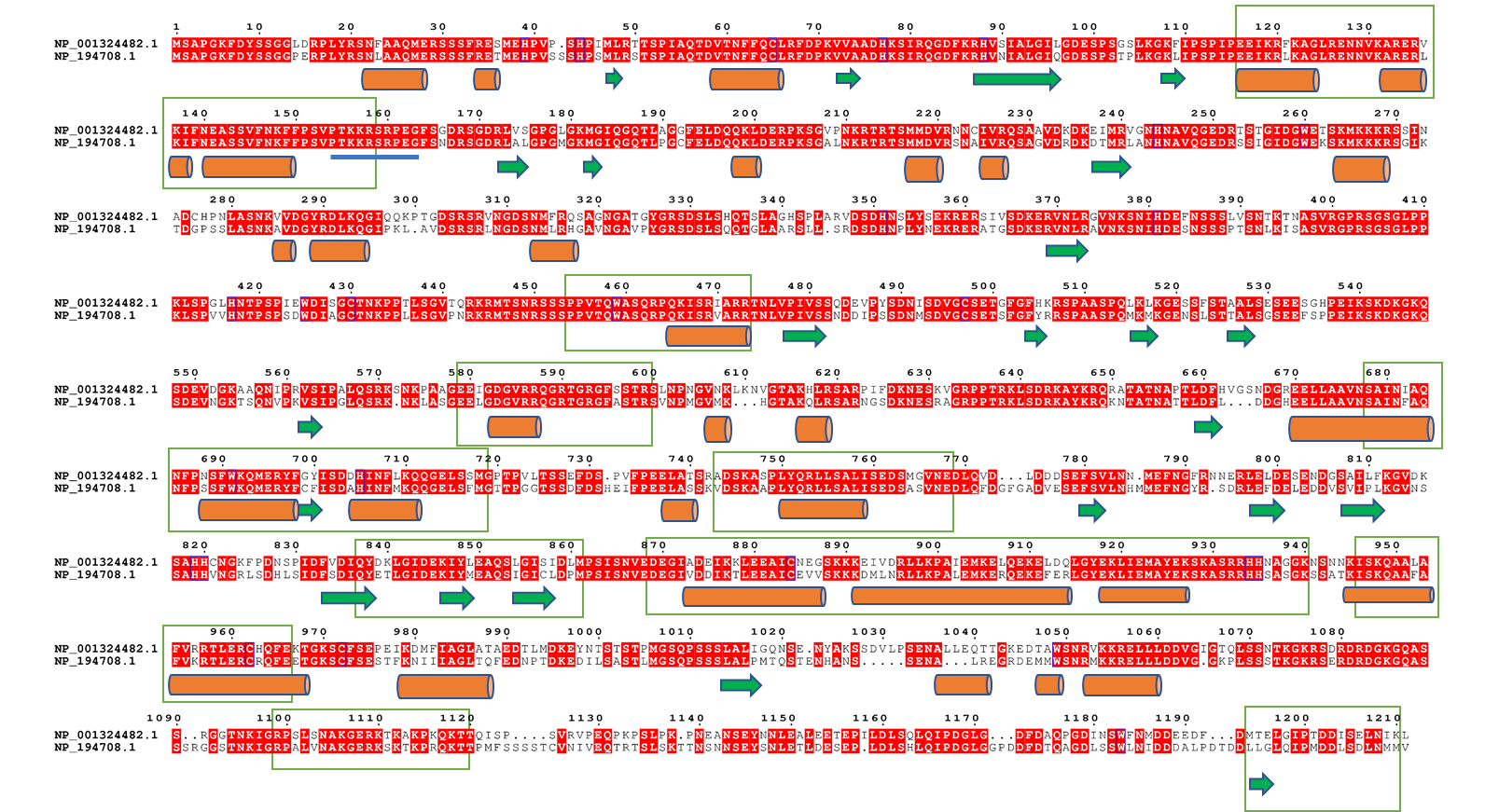


Figure S2. Secondary structure of Arabidopsis ADA3 proteins. Alignment of *Arabidopsis thaliana* ADA3a (NP_001324482.1) and ADA3b (NP194708.1) proteins. Conserved amino acids are in white with red background. Secondary structure prediction shows b-sheets with green arrows and a-helices with red cylinders. The nuclear localization signal is shown with blue line and the predicted motifs are shown in boxes.


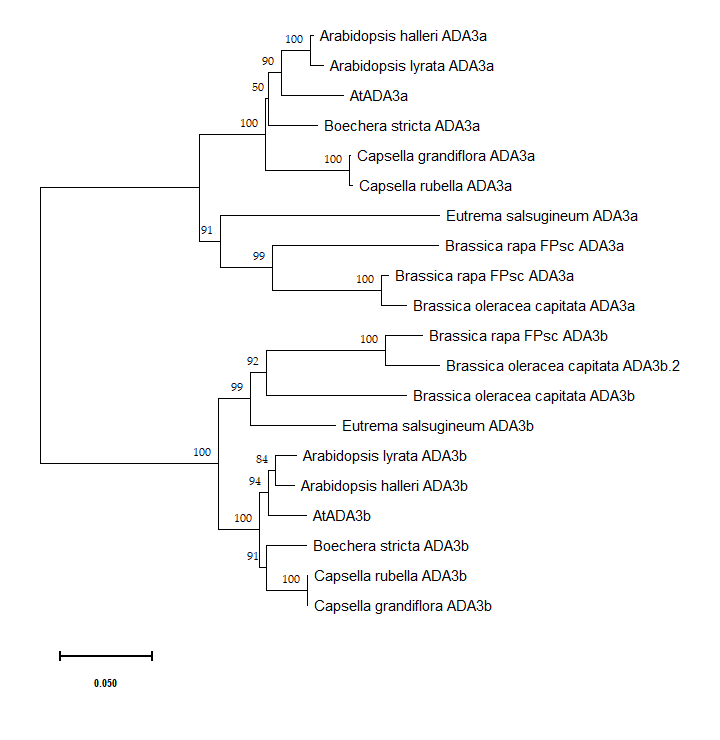


Figure S3. ADA3 proteins from species of Brassicaceae family.


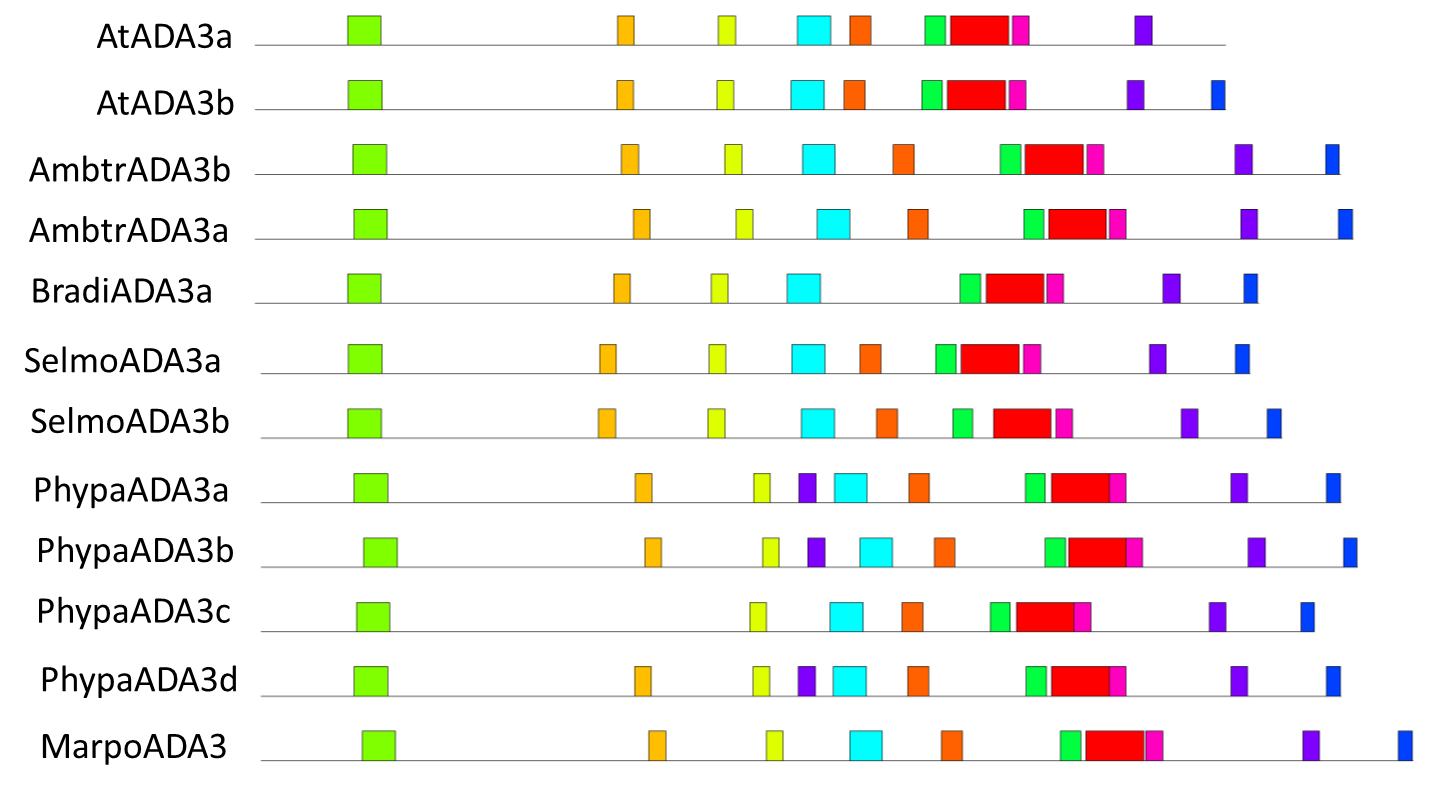


Figure S4. Discovery of Amino acids Motif in ADA3 proteins using MEME algorithm (http://meme.nbcr.net). The ten best motifs is displayed with boxes along the primary amino acid sequence of plant ADA3 proteins. The motif sites has a p-value ≤ e-10


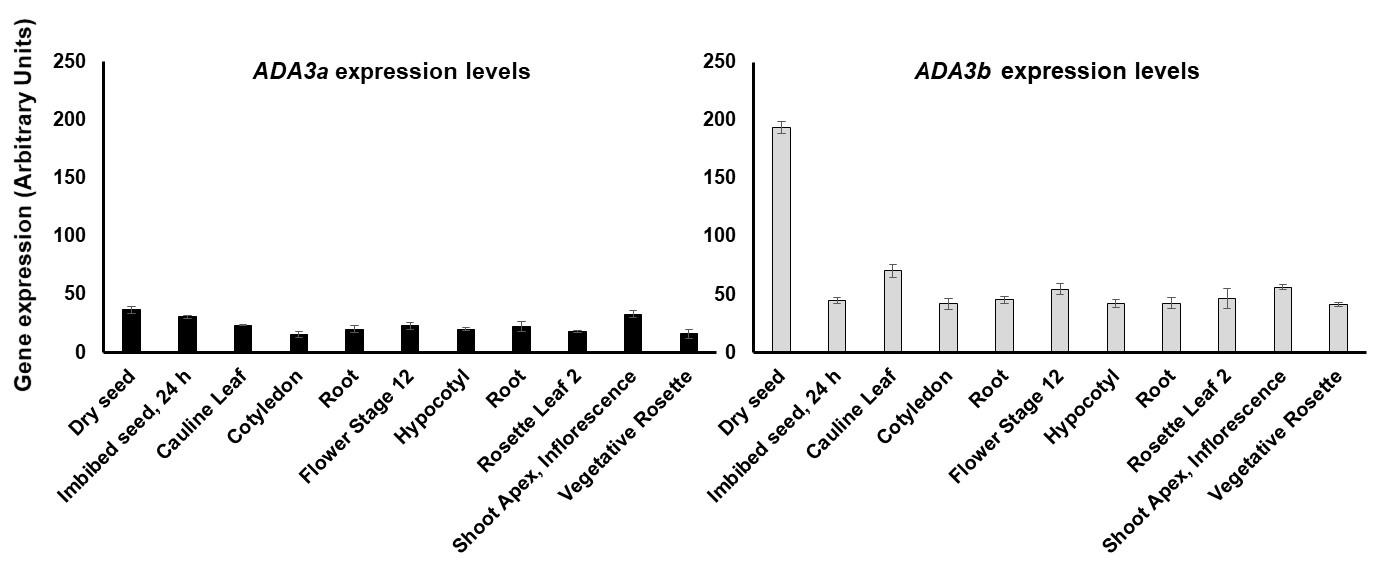
Figure S5. mRNA expression levels of ADA3a and ADA3b in various organs and tissues, using the eFP browser.


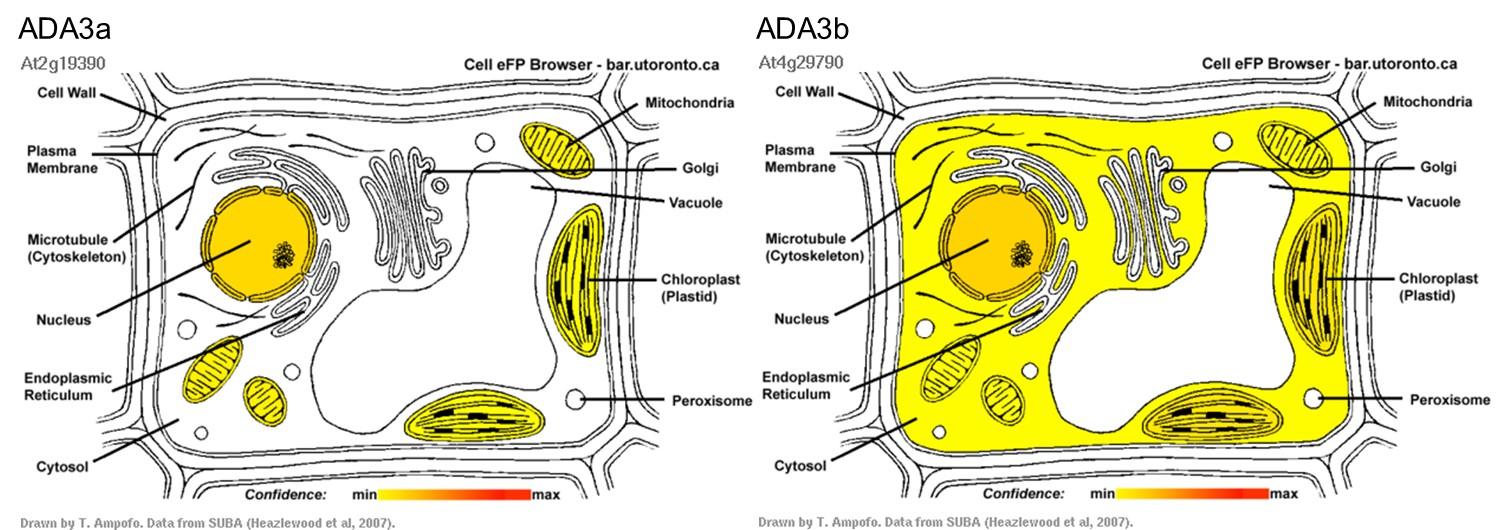


Figure S6. Predicted sub-cellular localization of ADA3a and ADA3b proteins using the Cell eFP browser.


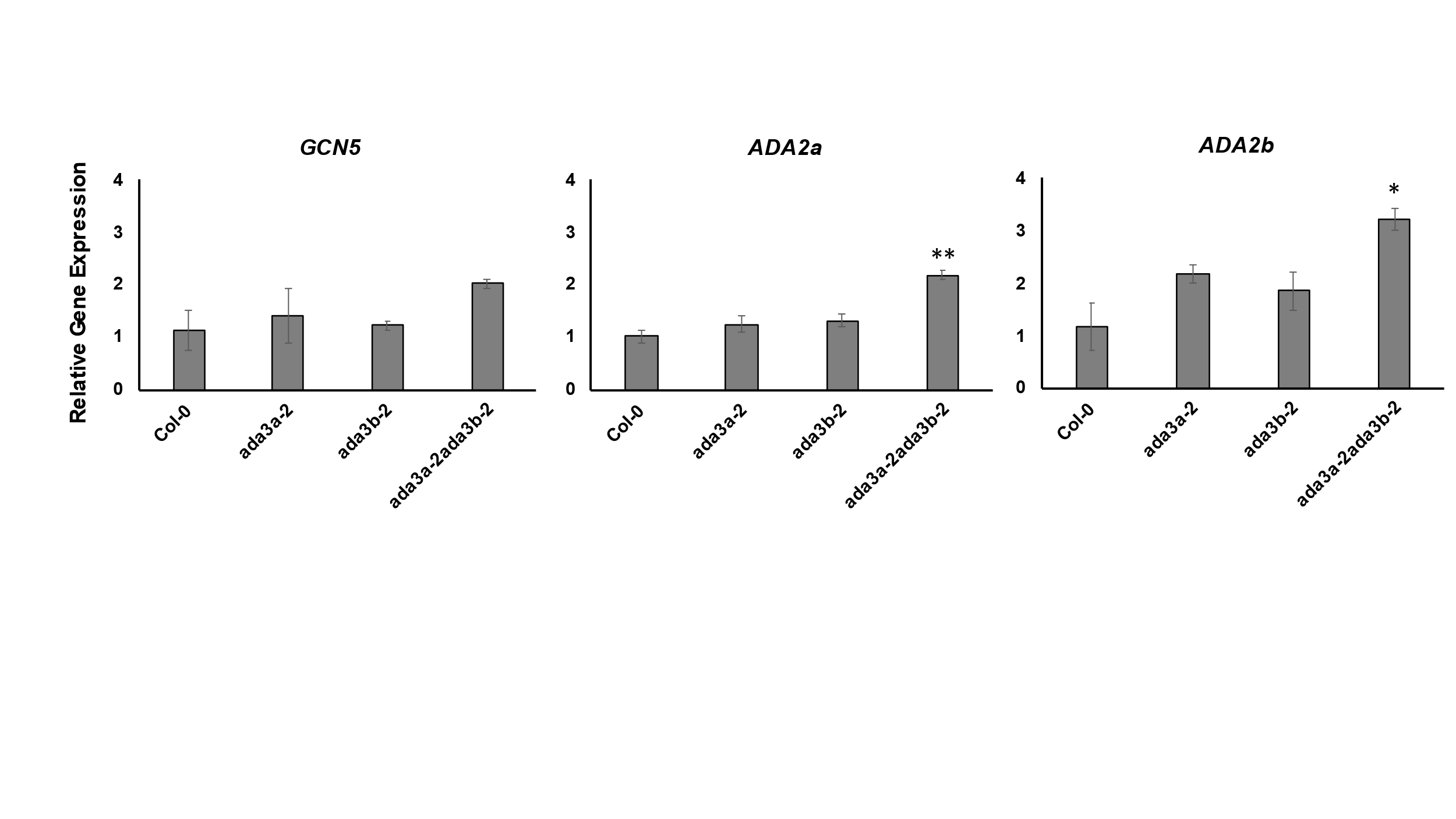
Figure S7. The effect of ada3a-2 and ada3b-2 mutants on the expression of GCN5, ADA2a and ADA2b genes. The error bars represent standard error (SE) of three technical repeats. Statistical significance calculated by t-test, *P<0.05, **P<0.01, ***P<0.001.


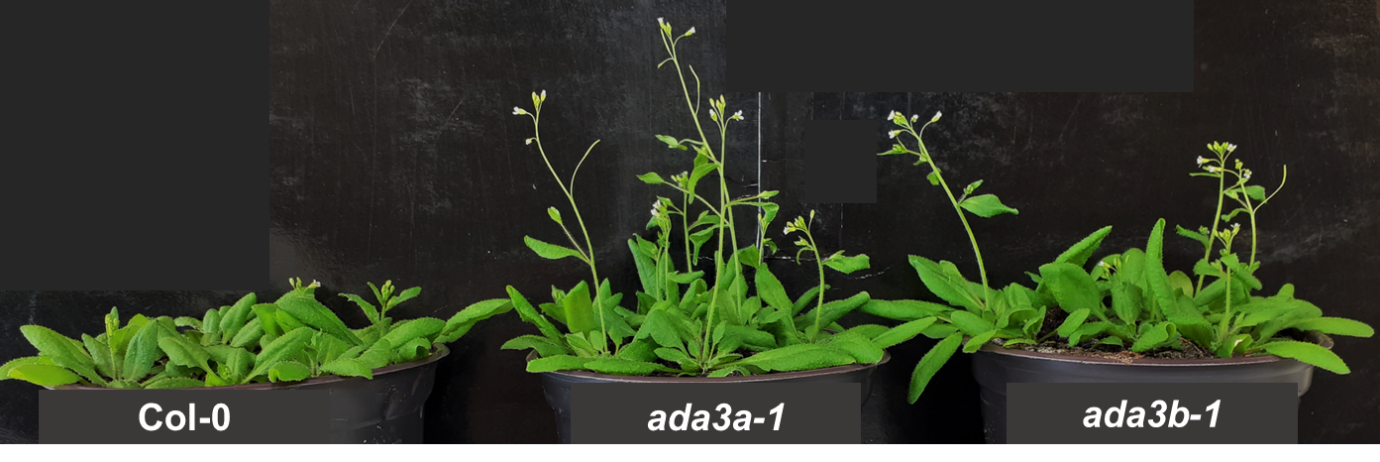


Figure S8. Flowering phenotypes of *ada3a-1* and *ada3b-1* mutant plants grown under LD conditions


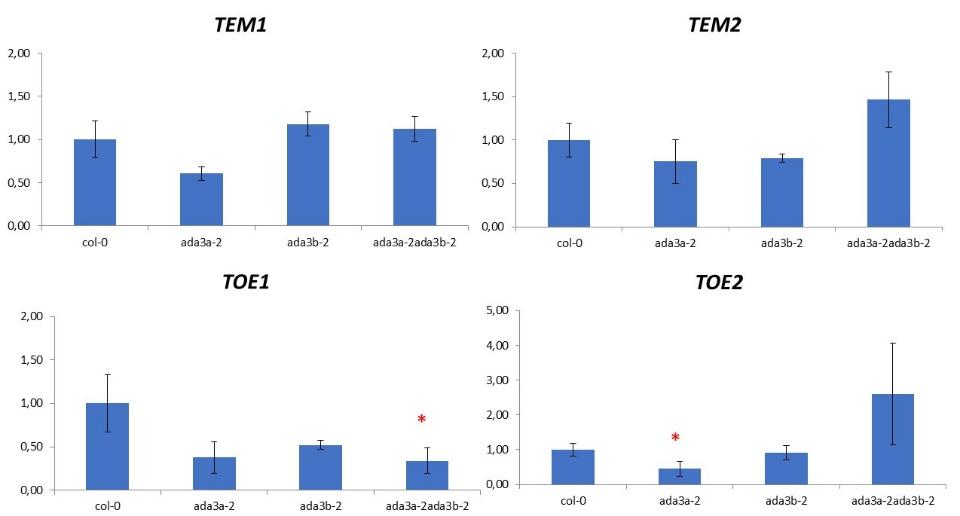


Figure S9. Gene expression of flowering related genes
